# Decapod Crustaceans in a Transitional River System: Insights from the Ribeira de Iguape Ecoregion, Brazil

**DOI:** 10.3390/biology14091255

**Published:** 2025-09-12

**Authors:** Esthephany Konesuk Santos Miranda, Maria Rosa Roque Santana Gomes, Luis Carlos Ferreira de Almeida, Giovana Bertini

**Affiliations:** FCAVR-School of Agricultural Sciences of Vale do Ribeira “Registro Campus”, “UNESP” State University of São Paulo, Av. Nelson Brihi Badur, 430, Vila Tupy, Registro 11900-000, SP, Brazil; esthephany.ks.miranda@unesp.br (E.K.S.M.); mrrs.gomes@unesp.br (M.R.R.S.G.); luis.almeida@unesp.br (L.C.F.d.A.)

**Keywords:** amphidromous shrimp, community dynamics, non-native species, seasonal variation

## Abstract

Freshwater ecosystems are essential for biodiversity conservation but face increasing threats from human activities. This study assessed the species richness, diversity, and spatial and temporal distribution of decapod crustaceans (Caridea and Brachyura) in the lower Ribeira de Iguape River, southeastern Brazil, a globally recognized freshwater ecoregion. Monthly sampling took place over one year at three sites using both active and passive capture methods. Environmental parameters were measured in the field, and multivariate analyses (correspondence analysis, PERMANOVA, and multiple regression) were applied to explore species–environment relationships and community structure. A total of 42,897 individuals were recorded, including eight Caridea and four Brachyura species. Carideans accounted for over 97 percent of the total, with *Potimirim* spp. and *Macrobrachium acanthurus* as the most abundant. The site nearest the river mouth showed the highest species richness and diversity, likely due to its transitional characteristics and moderate salinity variation. Environmental variables such as dissolved oxygen and salinity influenced species occurrence, although biological interactions may also contribute. Seasonal changes in abundance appeared most evident during winter and spring, driven by shifts in dominant taxa. The presence of the non-native *Macrobrachium rosenbergii* suggests early establishment and reinforces the need for long-term ecological monitoring in transitional freshwater environments.

## Simple Summary

This study examines the diversity and distribution of shrimp and crabs in a river region where freshwater meets the sea, in southeastern Brazil. Monthly collections were carried out over one year in three different areas of the Ribeira de Iguape River, using hand nets and traps. These methods helped capture species living in different habitats along the river. The survey recorded more than 42,000 individuals, comprising twelve species. Most were freshwater shrimp, and one species, originally from another region, was identified for the first time in this area. This finding highlights the importance of continuous monitoring. Two shrimp species were especially abundant, each dominating different sections of the river. The area near the river mouth had the highest number of species, likely due to its transitional nature and the influence of both fresh- and saltwater. The total number of individuals varied by season, with greater numbers observed in winter and spring. Although environmental factors were related to species presence, other biological aspects not addressed in this study may also influence the distribution of these animals. These results significantly contribute to a better understanding of river ecosystems and provide valuable insights to support efforts in conserving biodiversity.

## 1. Introduction

Freshwater environments cover approximately 0.8% of the Earth’s surface but harbor at least 6% of all species described [1]. However, these ecosystems are among the most threatened, experiencing significant biodiversity loss due to several threats, including dam construction, dredging, pollution, the introduction of non-native invasive species, and intensive agricultural practices [2,3,4,5].

In the southeastern region of São Paulo State, the Ribeira de Iguape River basin plays a fundamental role in the socioeconomic dynamics of Vale do Ribeira communities, where artisanal fishing is particularly prominent. The Cananéia–Iguape lagoon system stands out as a central fishing hub, with activities concentrated in the Ribeira River and primarily targeting species such as manjuba (Engraulidae: anchovy), robalo (Centropomidae: snook), and the freshwater shrimps *Macrobrachium acanthurus* and *Macrobrachium carcinus*, which are harvested for use as live bait and for human consumption, respectively [6,7,8,9,10,11,12,13,14]. Human activities strongly impact this basin and have suffered environmental degradation in recent decades due to unregulated mining [15]. As a result, residues containing heavy metals remain accumulated in the river sediments throughout its course to the estuarine region [16,17]. Additionally, much of the riparian vegetation has been replaced by banana plantations and pastures [18], leading to the release of agrochemicals such as carbamates and glyphosate-based herbicides, among others, into aquatic environments [19,20]. However, there is a notable lack of ecotoxicological studies specifically addressing the impacts of these contaminants on key native species within the Ribeira de Iguape basin.

In addition to land-use impacts and water contamination, the introduction of non-native species represents another significant threat to this ecosystem. A notable example of freshwater crustacean dispersal due to human activities in Brazil is the presence of the giant freshwater prawn (*Macrobrachium rosenbergii*) in the eastern Amazon region and the state of Espírito Santo [21,22]. In São Paulo State, although there is no evidence of successful reproduction in natural environments to date, escapes of individuals from aquaculture farms have been reported [23].

The “Ribeira de Iguape region” is recognized by WWF as an ecoregion due to its ecological uniqueness and importance for aquatic biodiversity [24]. This area contains a critical freshwater reserve in São Paulo State and one of Brazil’s best-preserved genetic reservoirs [25]. The Vale do Ribeira includes 45 active conservation units across its territory [26]. It also retains more than 50 percent of the remaining native vegetation of the Atlantic Forest in Brazil [27], underscoring the essential role of these protected areas in conserving this biome. By the late 1990s, researchers had already stressed the need to intensify studies and collection efforts to better document species richness in São Paulo State, identifying the Vale do Ribeira as a priority region [28].

Although recognized as a conservation priority, the region still lacks comprehensive data on the biodiversity and distribution patterns of aquatic fauna, which limits the ability to predict ecological impacts and develop effective mitigation strategies. Notable studies on crustaceans include a compilation of freshwater decapod diversity in São Paulo State that listed 32 species from five families and eight genera [29]; an analysis of the high phylogenetic diversity among anomurans of the genus *Aegla* in the Ribeira do Iguape ecoregion, which highlighted the need for targeted conservation of these organisms and their habitats [30]; and a survey of decapod species richness and distribution across seven conservation areas in the Vale do Ribeira, which documented nine caridean shrimp species, five aeglid anomurans, and four brachyurans [31]. Additional studies have explored the biology and ecology of freshwater shrimp, including *M. acanthurus*, *M. carcinus* [32,33,34,35,36], and *Macrobrachium olfersii* [37,38].

Thus, despite having high species richness, significant endemism, and vulnerability, freshwater ecosystems still receive limited attention in large-scale conservation efforts [24]. This limited focus is partly due to the lack of comprehensive and organized data on the distribution of freshwater species [39]. Considering this, the present study assesses the species richness, diversity, and spatial and temporal distribution of the decapod crustacean community (Caridea and Brachyura) in the lower course of the Ribeira de Iguape River. The findings support management plans and conservation strategies for species and their habitats, enable the detection of non-native species, and help maintain the ecosystem’s integrity and stability.

## 2. Materials and Methods

### 2.1. Study Area

The Ribeira de Iguape River basin (24°42′29″ S; 47°33′19″ W) is one of the largest along the Brazilian coast, covering about 25,000 km^2^ of drainage area, of which 17,068 km^2^ lie within the state of São Paulo, covering 28 municipalities. Its headwaters are in the state of Paraná, and the river flows for about 470 km before reaching the Atlantic Ocean in the city of Iguape, on the southern coast of São Paulo State (SP) [40,41]. Near the coastal region, the river delivers a high freshwater load to the estuarine environment, forming the Cananéia–Iguape system, a network of lagoon channels situated within the United Nations Educational, Scientific and Cultural Organization (UNESCO) Biosphere Reserve [42].

The region is classified as having a humid subtropical climate according to the Köppen climate classification system [40]. Based on data from the automatic meteorological station (A712) of the National Institute of Meteorology (INMET, https://bdmep.inmet.gov.br/ accessed on 28 July 2025) in the city of Iguape, collected between April 2014 and January 2015, the average annual precipitation was 1267 mm, and the mean temperature was 21.4 ± 3.3 °C, ranging from 13.8 °C to 34.4 °C across seasons.

In the mid-18th century, a significant hydrological alteration occurred in the Ribeira de Iguape River, leading to the construction of the Valo Grande Channel near the urban center of Iguape [13]. This intervention diverted approximately 60% of the river’s flow into the lagoon system, causing a significant drop in salinity levels [13,40,42,43]. Consequently, the region is characterized by moderate tidal influence, with current velocities reaching 0.6 m/s during flood tides and 0.7 m/s during ebb tides [44]. This anthropogenic alteration also led to a decline in the diversity of fishery resources in the town of Iguape, compared to the neighboring city of Cananéia, where marine species still dominate local fisheries. In Iguape, by contrast, fishing has become increasingly based on freshwater species due to the reduced salinity levels [13].

### 2.2. Collection of Specimens and Environmental Parameters

**Figure 1:**
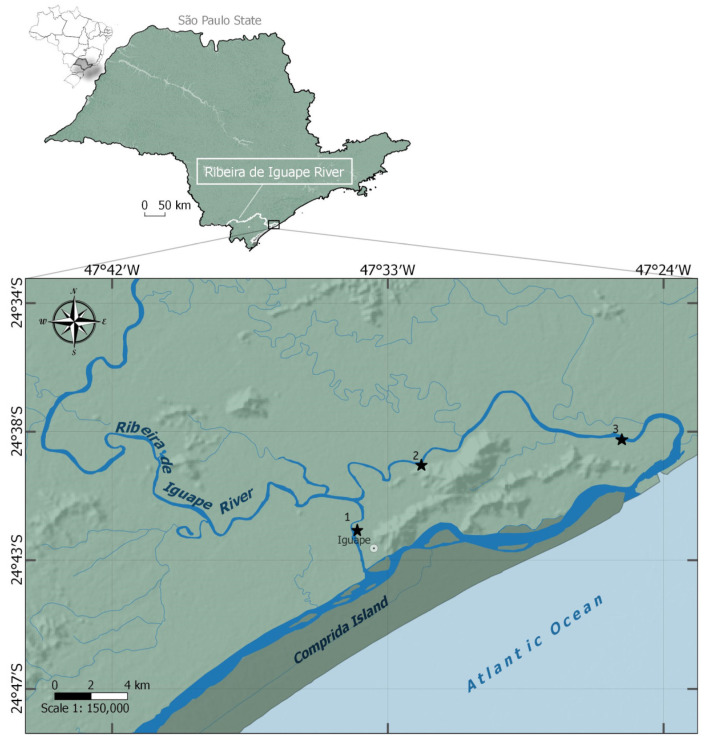
Map of the Ribeira de Iguape River basin, São Paulo, Brazil, showing the decapod community sampling areas (1, 2, and 3) from 14 February to 15 January in the Iguape region.

From February 2014 to January 2015, monthly sampling was conducted during low tides in three areas along the lower course of the Ribeira de Iguape River, in Iguape, São Paulo State, Brazil. The first sampling area was located on the Valo Grande Channel, which is 250 m wide and 7 m deep. In contrast, the second and third areas were located along the river’s natural course, approximately 15 km and 8 km from the river mouth, respectively (Figure 1). Although the data were collected nearly a decade ago, there is no indication of anthropogenic changes or land-use alterations in the region during this period. Ongoing research efforts by the authors in the area have not revealed substantial shifts in environmental conditions nor the implementation of new conservation policies. Therefore, the dataset remains a valuable and representative baseline for characterizing this poorly studied estuarine system.

No riparian forest is present along the riverbanks at any of the three sampled sites. In Area 1, the bank consists of a mixture of grasses (*Brachiaria* spp.) and aquatic plants, including floating water hyacinth (*Eichhornia crassipes*). In Area 2, aquatic plants are the most common, and filamentous algae appear at certain times of the year. In Area 3, grasses are the dominant vegetation.

The collections aimed to provide both qualitative and quantitative assessments of the decapod fauna through passive and active capture methods, applied monthly across three areas over a 12-month period, totaling 36 standardized events. This methodological approach follows the procedures established by [36,37], and was designed to minimize sampling bias by targeting different ecological niches and body size classes within the decapod community. As an active method, a sieve measuring 0.9 m in diameter with a 3 mm mesh size was used. Two people swept the sieve through partially submerged marginal vegetation for 20 min to capture individuals associated with this vegetation. Additionally, twelve traps were employed for passive sampling: six minnow traps and six box traps. The minnow traps (1.0 m long, 0.5 m in diameter, and 8 mm mesh size) were placed on the riverbed to capture bottom-dwelling individuals. The box traps (0.55 m long × 0.36 m wide × 0.25 m high), covered with 8 mm mesh netting and equipped with two lateral openings of 6 cm in diameter, were positioned and tied beneath the marginal vegetation. All traps were left in the water for approximately 24 h, baited with fish remains and cow bones.

At each collection site, data on water temperature (°C), salinity (ppt), pH, dissolved oxygen (mg L^−1^), and conductivity (µS cm^−1^) were obtained using a multiparameter probe (YSI-ProPlus, made in Yellow Springs, OH, USA) inserted into the water column approximately 30 cm below the surface. This depth is considered adequate for establishing relationships between environmental parameters and the decapod community, as previously applied in estuarine floodplain studies in the Brazilian Amazonia [45]. The transparency of the water was checked with a Secchi disk with a cable graduated in centimeters.

After capture, the animals were transported to the laboratory and frozen until further processing. Species identification followed established taxonomic references [46,47,48]. Shrimps of the genus *Potimirim* were abundant in the collections, making it impractical to identify and measure all individuals. Therefore, identification and morphometric analyses were performed during randomly selected months to determine species within this genus, while considering the total number of individuals in the subsequent analyses.

To evaluate shrimp size, subsampling was conducted on the total number of individuals collected for each species, by area and month. All individuals were examined in samples containing up to 80 specimens. If samples exceeded this number, a random subsample was taken, following an adaptation of established procedures [36,37], as follows: 80 individuals were selected from samples with 80–160 shrimps; 50% of individuals were used from samples with 160–320; 25% from samples with 320–500; and 10% from samples with more than 500 individuals. Carapace length (CL) was measured as the distance from the orbital angle to the posterior margin of the carapace. All Brachyura specimens were fully analyzed, with measurements taken at the carapace width (CW). A caliper with a precision of 0.01 mm was used for all measurements.

### 2.3. Data Analysis

#### 2.3.1. Environmental Factors

A generalized linear model (GLM) was used to evaluate the effects of sampling area and season (summer: January to March; autumn: April to June; winter: July to September; spring: October to December) on environmental parameters. Model selection was based on the Akaike Information Criterion (AIC), choosing the model with the lowest AIC value [49]. For temperature, pH, and dissolved oxygen, a normal distribution with an identity link function was applied. For salinity, conductivity, and water transparency, a gamma distribution with a log link function was used. All analyses were performed using IBM SPSS Statistics^®^ (trial version, Armonk, NY, USA).

#### 2.3.2. Community Analysis

##### Diversity, Evenness, and Species Richness

Species richness was measured by counting the number of species in the study area and the total abundance of individuals recorded in the samples [50]. Species diversity (H′) was calculated using the Shannon–Wiener index [51], which considers both species richness and the relative abundance of each species [52]. Evenness (J′) was determined using a method that accounts for the level of uniformity in species distribution [53,54]. This index ranges from 0 to 1, with values closer to 1 indicating a more even distribution of individuals among species [52]. All analyses were performed using the free software PAST (version 3.25, Oslo, Norway).

##### Multivariate Analysis

Correspondence analysis (CA) was employed to link total species abundance to the sampling areas across the entire study period. In this analysis, absolute abundance values were used, considering each species as an independent data group, which minimized the impact of the sampling design [55]. This analysis was conducted using the freely available software PAST (version 3.25).

A two-way permutational multivariate analysis of variance (PERMANOVA) was applied to compare the total abundance of decapods and the most abundant Caridea and Brachyura species across areas and seasons. This analysis was performed using the PC-ORD software (version 6, Gleneden Beach, OR, USA) [56].

Multiple linear regression analyses were used to examine how environmental variables affected the relative abundance of the most common decapod species. The predictor variables included water temperature, dissolved oxygen, electrical conductivity, salinity, pH, and water transparency. The dependent variables represented the most prevalent decapod species. Predictor variables were selected based on entry probability ≤ 0.05 and removal probability ≥ 0.10. Model fit was assessed using the adjusted R^2^, the F-value for overall significance, and *p*-values < 0.05. Standardized coefficients (Beta) indicated each predictor’s relative contribution, and tolerance values were examined to verify multicollinearity, with no serious issues detected [57]. All analyses used IBM SPSS Statistics^®^ (trial version).

## 3. Results

### 3.1. Environmental Factors

**Figure 2:**
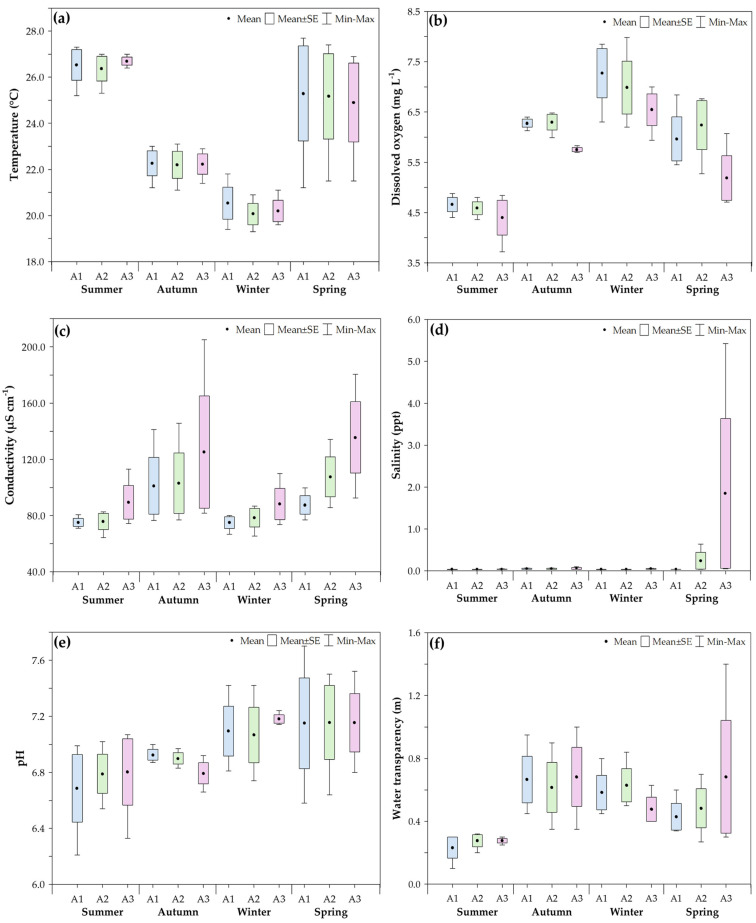
Boxplots showing the seasonal variation in environmental parameters ((**a**). water temperature; (**b**). dissolved oxygen; (**c**). electrical conductivity; (**d**). salinity; (**e**). pH; (**f**). water transparency) recorded in the three sampling areas from February 2014 to January 2015 in the Ribeira de Iguape River, Iguape (SP). A1 = Area 1, A2 = Area 2, A3 = Area 3.

The seasonal and spatial variation in the mean values of environmental parameters is illustrated in Figure 2. The results from the generalized linear model (GLM) showed a significant interaction between sampling area and season only for salinity (Wal χ^2^ = 32.17; *p* < 0.05). This interaction was primarily driven by the higher mean salinity observed in Area 3 during spring (0.71), which was significantly different from all other area–season combinations. Additionally, significant differences were found between areas for dissolved oxygen (Wald χ^2^ = 10.748; *p* < 0.05) and conductivity (Wald χ^2^ = 8.401; *p* < 0.05). Concerning seasonal variation, temperature (Wald χ^2^ = 98.551; *p* < 0.05), dissolved oxygen (Wald χ^2^ = 111.520; *p* < 0.05), conductivity (Wald χ^2^ = 18.482; *p* < 0.05), pH (Wald χ^2^ = 3.477; *p* < 0.05), and water transparency (Wald χ^2^ = 33.565; *p* < 0.05) also exhibited significant differences (Table 1).

### 3.2. Community Structure

A total of 42,897 individuals from the infraorders Caridea and Brachyura were collected, representing 12 species. Caridea was the most common group, making up 97.4% of all decapods (41,764 individuals), including members of the families Atyidae and Palaemonidae. Brachyura consisted of 1133 individuals (2.6% of the total), spread across four families: Sesarmidae, Portunidae, Panopeidae, and Ocypodidae. The complete species list, along with the total number of individuals and their minimum, maximum, and average sizes (mm), is shown in Table 2.

**Figure 3:**
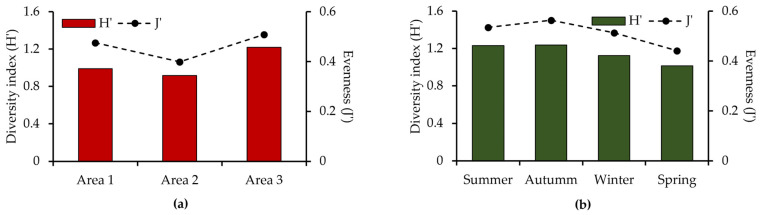
Spatial and temporal variation in the diversity index (H′) and evenness (J′) from February 2014 to January 2015 in the Ribeira de Iguape River, Iguape (SP). (**a**) Variation by area; (**b**) variation by season.

The overall diversity index for the region was 1.12 bits per individual, with an evenness value of 0.47. Among the sampled areas, Area 3 had the highest species richness (11 species), diversity index (H′ = 1.22), and evenness (J′ = 0.51) (Figure 3a). Seasonally, summer and autumn showed the highest diversity and evenness values compared to other seasons (Figure 3b).

Among the carideans, the family Atyidae accounted for 48.5% of the total shrimp collected, including *Potimirim potimirim* and *Potimirim brasiliana*, collectively called *Potimirim* spp. The family Palaemonidae was represented by five species: four from the genus *Macrobrachium* and one from the genus *Palaemon*. *Macrobrachium acanthurus* had the second highest abundance among the carideans (41.4%), followed by *Palaemon pandaliformis* (6.6%), *Macrobrachium olfersii* (3.4%), and *Macrobrachium carcinus* (0.1%). The non-native species, *Macrobrachium rosenbergii*, was represented by two individuals (0.005%) (Table 3).

Five species of brachyurans were observed, belonging to the genera *Armases*, *Callinectes*, *Minuca*, and *Panopeus*. The most common species was *Armases rubripes* (87.2%), followed by *Callinectes sapidus* (7.6%), *Callinectes bocourti* (3.8%), *Panopeus rugosus* (1.3%), and *Minuca mordax*, which was represented by a single individual (0.1%) (Table 3).

Among the sampling methods used, active capture with a sieve was the only effective way to sample *Potimirim* spp. This method was also the most efficient for catching the shrimps *P. pandaliformis*, *M. acanthurus*, *and M. olfersii*, as well as the crab *A. rubripes*. In contrast, passive methods (box and minnow traps) were effective in capturing larger-bodied shrimps and brachyuran crabs (Table 3).

**Figure 4:**
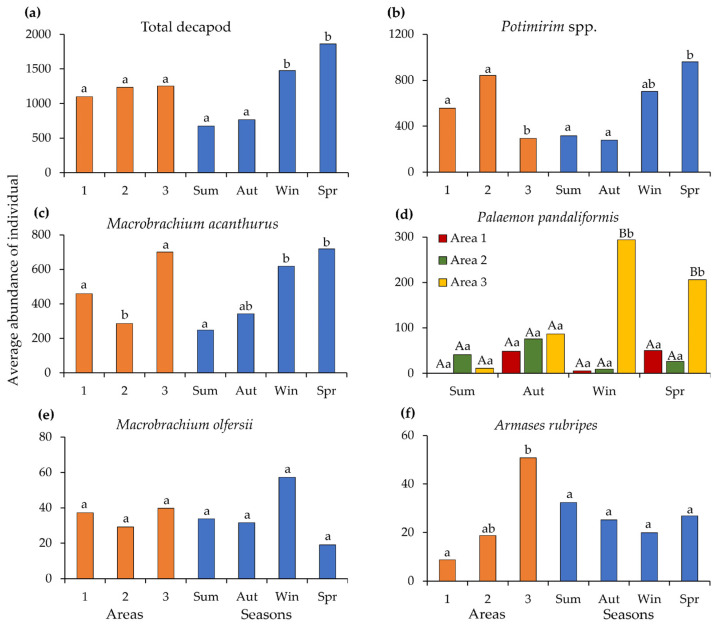
The results of the permutational multivariate analysis of variance (PERMANOVA) for the total number of decapods and the most abundant species, examining the effects of sampling area and season and their interaction: (**a**) total decapod; (**b**) *Potimirim* spp.; (**c**) *Macrobrachium acanthurus*; (**d**) *Palaemon pandaliformis*; (**e**) *Macrobrachium olfersii*; (**f**) *Armases rubripes*. (Sum—summer, Aut—autumn, Win—Winter, Spr—spring). Since no significant interaction between area and season was found, identical lowercase letters between columns of the same color (area or season) indicate no statistically significant differences (*p* > 0.05). For *Palaemon pandaliformis*, where a significant interaction between area and season was observed, capital letters indicate significant differences among areas within each season, whereas lowercase letters indicate significant differences among seasons within each area (*p* < 0.05).

The composition and total abundance of decapod species by area and season are shown in Table 3. Overall decapod crustacean abundance varied only across seasons (PERMANOVA; Pseudo F = 6.93, *p* < 0.05) (Figure 4a). Among the carideans, the abundance of *Potimirim* spp. showed significant differences between areas (Pseudo F = 7.63, *p* < 0.05) and seasons (Pseudo F = 2.44, *p* < 0.05) (Figure 4b). A similar pattern was observed for *M. acanthurus* (areas: Pseudo F = 3.73, *p* < 0.05; seasons: Pseudo F = 3.27, *p* < 0.05) (Figure 4c). For *M. olfersii*, no significant differences were found for either factor (*p* > 0.05) (Figure 4d), whereas *P. pandaliformis* exhibited an interaction between area and season (Pseudo F = 1.76, *p* < 0.05), with a higher number of individuals recorded in Area 3 during winter and spring (Figure 4e). For the most abundant brachyuran, *A. rubripes*, the distribution differed only among areas (Pseudo F = 2.70, *p* < 0.05) (Figure 4f).

**Figure 5:**
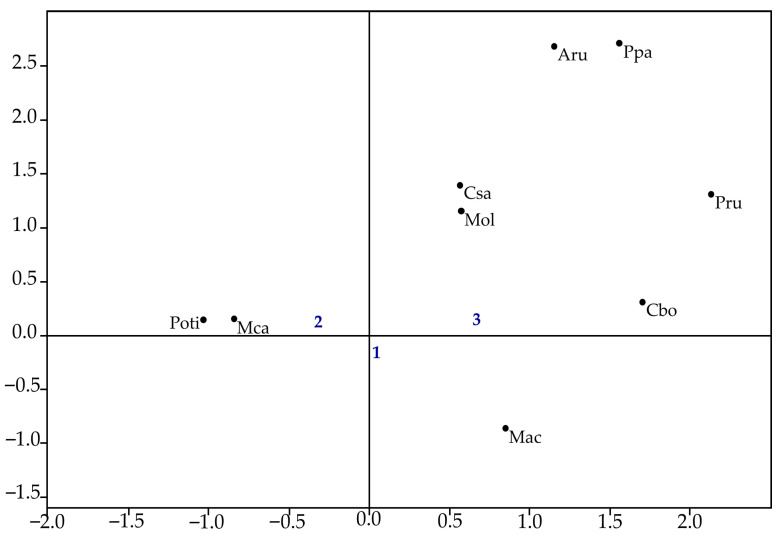
Correspondence analysis of decapod species abundance across sampling areas. (Mol—*Macrobrachium olfersii*, Aru—*Armases rubripes*, Ppa—*Palaemon pandaliformis*, Mac—*Macrobrachium acanthurus*, Poti—*Potimirim* spp., Mca—*Macrobrachium carcinus*, Cbo—*Callinectes bocourti*, Csa—*Callinectes sapidus*, Pru—*Panopeus rugosus*).

Correspondence analysis by area showed that axis 1 explained 53.6% of the total data variation, and axis 2 explained 26.9% (χ^2^ = 21.014, *p* < 0.05). The species *P. pandiformis*, *M. olfersii*, and all brachyurans showed a stronger association with Area 3, while *M. acanthurus* was more closely linked to Areas 1 and 3 (Figure 5).

The results of the multiple linear regression analyses showed that different environmental variables were significantly and positively linked to species abundance (Table 4). Dissolved oxygen levels were significantly associated with the abundance of *Potimirim* spp. (*p* = 0.013), while pH was the predictor for *M. acanthurus* (*p* = 0.004). Water salinity was identified as an explanatory variable for *M. olfersii*, *P. pandaliformis*, and *A. rubripes*. Although the adjusted coefficients of determination (adjusted r^2^) ranged from 13.7% to 21.5%, the models revealed trends in the relationship between water physicochemical conditions and species distribution. For *M. carcinus*, *C. bocourti*, *C. sapidus*, and *P. rugosus*, the stepwise method did not select any environmental variables for the models, indicating no statistically significant associations with the tested variables.

## 4. Discussion

This study advances environmental monitoring and biodiversity protection in the Vale do Ribeira region. Regional faunal surveys are crucial for understanding ecosystem structure and function, especially as pressures from human activities and climate change increase. Such studies are vital for developing environmental monitoring programs and provide a basis for effective biodiversity conservation efforts [59].

The results are consistent with previous studies that identified the Ribeira de Iguape ecoregion as a conservation priority due to its high concentration of genetic diversity within a single watercourse, the Ribeira de Iguape River [30]. This classification followed a conservation assessment framework that considered species richness, endemism, threat status, and both genetic and phylogenetic diversity across South American freshwater ecoregions [30]. The observed patterns in decapod crustacean diversity and distribution reinforce the region’s ecological importance and emphasize the need for sustained protection and responsible management.

Comparing species richness across different regions is complex, as species counts vary depending on factors such as sampling effort, area size, climate, and substrate types [60]. In freshwater habitats, the complexity increases due to the high internal diversity of these systems, which often requires multiple sampling methods, including active techniques (e.g., sieving and manual searching) and passive ones (e.g., traps), both of which directly influence the number of species recorded [61,62,63].

The combination of sieving and traps used in this study, designed to target the specific niches occupied by different species, allowed for a broader understanding of decapod composition in the region, as each method was essential for capturing different species. In a study on the diversity and biology of swimming crabs (Portunidae) in the Estuarine–Lagoon Complex of Iguape, Ilha Comprida, and Cananéia, ten species were documented, including *Callinectes sapidus* and *Callinectes bocourti*, which were also found in the present study, reinforcing the estuarine influence on the lower course of the Ribeira de Iguape River [64]. However, surveys specifically focused on brachyuran composition in the region, especially in the Ribeira de Iguape River, remain limited. A previous study conducted in the Ribeira de Iguape basin and neighboring coastal areas did not include sampling within the river itself [31], which may explain the lack of records for species such as *Armases rubripes*, *Minuca mordax*, and *Panopeus rugosus*. These species are reported here for the first time in this area. Although primarily marine, these crabs are commonly found in brackish environments like lagoons and estuaries, and they may also be present in freshwater systems, including river deltas, during certain life stages. This distribution pattern reflects their ability to tolerate variations in salinity and temperature [65,66].

Regarding the carideans, all Atyidae and Palaemonidae species previously recorded in headwater and mid-course areas of the Ribeira de Iguape River basin [31], including *Potimirim potimirim*, *Potimirim brasiliana* (=*Potimirim glabra*), *Macrobrachium acanthurus*, *Macrobrachium olfersii*, and *Macrobrachium carcinus*, that were also found in the present study, except *Macrobrachium heterochirus* and *Macrobrachium potiuna*, which were absent because they are associated with headwater habitats characterized by rocky substrates and better-preserved riparian vegetation.

Among the species recorded in this study, *M. carcinus*, *M. acanthurus*, and *C. sapidus* warrant special attention for conservation, as reflected in their listing in the Livro Vermelho dos Crustáceos do Brasil [Red List of Brazilian Crustaceans] [58]. These species are categorized as Data Deficient (DD), indicating a lack of reliable data on their population status and calling for detailed assessments of their population trends, particularly given their commercial value. National action plans aimed at protecting aquatic and mangrove-associated fauna also identify these species as conservation priorities. The other species recorded in this study are classified as Least Concern (LC), suggesting that their populations are currently stable. Nevertheless, their inclusion on the Red List reinforces the need for continuous monitoring, especially in environments subject to gradual changes that may compromise long-term species persistence.

The detection of *Macrobrachium rosenbergii*, a non-native species introduced to Brazil in the 1970s for aquaculture, remains significant due to its ongoing spread [23,67]. Its presence has been recorded in 43 municipalities across eight Brazilian states, with the most occurrences reported in natural environments in Pará [68,69,70,71]. In São Paulo, only one previous record exists in natural waters, documenting a single specimen in the Tietê River basin. In the Vale do Ribeira region, the cultivation of this species was authorized in 1993 in the Etá River (Eldorado, SP), a tributary of the Ribeira de Iguape River [72], which may have been the initial introduction point along São Paulo’s southern coast. Recent sampling efforts in the Iguape region (February 2025) recorded small individuals (12.4 and 24.5 mm CL; Bertini, pers. comm.), and local fishers frequently report capturing the species, including ovigerous females, indicating that its presence may be more widespread than previously documented. Despite the low abundance observed, this population may be in the early stages of establishment, possibly limited by competition with native species such as *M. acanthurus* and *M. carcinus*, which exhibit highly territorial behavior. In northern Brazil, reproductive populations are already established, with records of ovigerous females in estuarine habitats, the identification of juveniles through molecular analyses, and the occurrence of all male morphotypes, indicating successful reproduction and recruitment in the wild [73]. The species also shows higher fecundity than native prawns, territorial behavior, and omnivorous feeding habits, which may contribute to competition for space and food resources [73]. The occurrence of *M. rosenbergii* in transitional zones such as the lower Ribeira de Iguape River reinforces the need for continuous monitoring of non-native species and their potential consequences for native communities.

The environmental variations observed in the lower course of the Ribeira de Iguape River result from the combined influence of saltwater intrusion and the region’s rainfall regime. Although saline water reaches this part of the river, the high freshwater flow limits the progression of the salt wedge, especially during the rainy season. Salinity ranged from nearly zero to five, with the highest levels recorded in Areas 2 and 3 during spring, the season with the least recorded rainfall (331.8 mm, INMET). A similar trend was observed in electrical conductivity, which rose due to the higher concentration of dissolved ions associated with marine influence during this period. Despite the narrow salinity range, these values are consistent with the behavior of subtropical estuaries under strong freshwater influence, such as the Ribeira de Iguape River. The observed increases, especially in Area 3 during the drier months, indicate a localized but significant marine influence, enough to define this section as a transitional zone between freshwater and estuarine environments. The year-round presence of marine species, such as swimming crabs (Brachyura), in this area further supports this classification. This hydrological pattern resembles that observed in Guajará Bay, which, because of its high freshwater input from the Amazon River, acts like a river for most of the year, effectively preventing the salt wedge from advancing during the rainy season [45]. Similarly, the other physicochemical parameters recorded in the Ribeira de Iguape River, including temperature, pH, water transparency, and dissolved oxygen, follow patterns commonly reported for estuarine systems, where seasonal variation and proximity to the coast jointly influence environmental dynamics [45,74,75,76].

The seasonality observed in the total decapod community, with higher abundances during winter and spring, mainly reflects the population dynamics of the dominant species, especially the caridean shrimps. These patterns also influenced diversity indices, with the highest diversity and evenness observed in autumn and the lowest in winter and spring. These periods coincided with higher abundances of *M. acanthurus* and *Potimirim* spp., indicating that seasonal decreases in diversity are due to the numerical dominance of these taxa. Such patterns are consistent with the amphidromous behavior of *M. acanthurus*, which migrates upstream as individuals grow, as documented along more than 80 km of the Ribeira de Iguape River [36]. In this system, juveniles typically concentrate near the river mouth and gradually migrate upstream as they grow. This ontogenetic migration shapes the spatial distribution and size structure of the population. A similar longitudinal migration can be inferred for *Potimirim* spp., supported by studies in other river systems where individuals at different life stages are found along various parts of the river, consistent with an amphidromous life cycle [77].

Studies conducted in other tropical and temperate estuarine systems support the influence of seasonal fluctuations on decapod communities. In the Mundaú/Manguaba lagoon complex (Alagoas, Brazil), higher abundances of *M. acanthurus* and *Palaemon pandaliformis* were recorded during the rainy season, in association with environmental factors [74]. Similarly, long-term studies in the temperate Guadalquivir River estuary (Spain) showed significant seasonal and yearly changes in community composition, with peaks in abundance, diversity, and biomass during spring and autumn, mainly driven by fluctuations in temperature and salinity [76,78]. These findings indicate that seasonal environmental changes modulate the composition, dominance, and distribution of decapod species. Among the caridean shrimps with migratory behavior observed in the present study, such variations may directly influence recruitment, growth, and spatial distribution processes, resulting in seasonal fluctuations in community structure.

The spatial distribution of the community did not reveal significant differences in overall abundance across areas, but dominant species showed distinct patterns of occupancy. Area 3, situated near the river mouth and influenced by marine conditions, provided favorable environments for both marine and freshwater species to coexist. This area had the highest values of species richness, diversity, and evenness, with all recorded taxa present. In contrast, Area 2 exhibited lower diversity due to the numerical dominance of *Potimirim* spp. These patterns emphasize the ecological importance of Area 3 and suggest that spatial variation in community structure was closely related to species evenness.

The distribution of the dominant species, in turn, was associated with variations in environmental factors, particularly salinity, dissolved oxygen, and pH. Patterns of association between these species, environmental gradients, and sampling areas emerged from both the multiple linear regression models and the correspondence analysis. However, the regression models showed low coefficients of determination, indicating that the selected variables accounted for only part of the observed variation. A comparable pattern occurred in the Guajará River estuary (Amazon region, Pará, Brazil), where abiotic factors had only weak correlations with species distribution [45]. These findings indicate that community structure results from a dynamic interaction of abiotic, biotic, and habitat features, including vegetation cover and the availability of organic debris, which influence both local diversity and species abundance.

Among the species contributing most to the spatial patterns observed, *A. rubripes*, *P. pandaliformis*, and *M. olfersii* stood out for their strong association with Area 3 and their positive relationship with salinity. Similar patterns have been documented in other regions of Brazil. In the case of *A. rubripes*, the association with salinity was evident in the salt marshes of the Patos Lagoon in southern Brazil, where ovigerous females occurred in greater numbers during periods of elevated salinity [79]. In addition, environmental features such as the presence of grasses in the marginal vegetation appear to favor the occurrence of this species in estuaries [80], a pattern also observed in the present study, as Area 3 had a high abundance of *Brachiaria* spp. For *P. pandaliformis*, its salinity-influenced distribution aligns with studies indicating a preference for estuarine environments [81,82]. However, no significant correlation was reported between this species and salinity in areas ranging from 2 to 9 ppt [83]. This divergence may reflect the high salinity tolerance of its larvae, which can withstand ranges from 0 to 35 ppt [84]. Salinities around 28 ppt are considered ideal for larval development, although lower values are necessary to trigger the process [84], which is compatible with the conditions observed in the sampled area. For *M. olfersii*, although adults are predominantly found in freshwater habitats, reproduction depends on brackish environments, as shown in previous studies [85,86]. Thus, salinity acts as a key factor for the presence of this species in transitional zones such as the one identified in the present study.

The distribution of *Potimirim* spp. showed a positive correlation with dissolved oxygen levels and was more abundant in Area 2, where greater coverage of filamentous algae and aquatic plants occurred. Such environments offer favorable conditions by providing shelter and food. According to [87], shrimps belonging to the family Atyidae use substrates covered with algae and decaying leaves as shelter. These surfaces host microbial biofilms, which the shrimps feed on by scraping or filtering particles, linking habitat selection directly to their feeding strategy. Higher algal biomass may also contribute to increased dissolved oxygen concentrations in the water, benefiting species that rely on well-oxygenated environments. In addition, the lower abundance of *Macrobrachium* species (such as *M. acanthurus* and *M. olfersii*) in Area 2 may reflect an avoidance mechanism by atyids, which may avoid areas with a higher risk of predation. This migration behavior in response to predator presence was demonstrated experimentally, with *Atya lanipes* responding to chemical and tactile cues from *M. carcinus* by altering its movement patterns and microhabitat use [88]. Similar results occurred in neotropical streams, where the presence of predatory fish inhibited the foraging activity of *P. glabra*, leading to the accumulation of periphyton [89]. These findings support the idea that non-consumptive, behaviorally mediated trophic interactions, such as predator avoidance, can influence the spatial distribution of atyid shrimps. This mechanism likely contributes to the higher abundance of *Potimirim* spp. observed in Area 2.

In the case of *M. acanthurus*, although a significant association with pH was identified, this parameter remained nearly neutral across all study areas, consistently within the optimal range of 6.5 to 8.5 for most *Macrobrachium* species [90]. Most individuals were collected using sieves in marginal vegetation, especially in Areas 2 and 3. This pattern suggests that the species’ distribution is better explained by the high abundance of riparian grasses, such as *Brachiaria* spp., rather than by pH itself, indicating a preference for this type of vegetative cover. These habitats provide protection from predators [64,91] and offer favorable conditions for feeding and reproduction [92]. The structure and composition of aquatic vegetation have been shown to influence decapod abundance and community composition in river systems of the Paraná floodplain, reinforcing the functional role of macrophytes in habitat selection [93]. Consequently, beyond abiotic conditions, the distribution of freshwater decapods also depends on biotic factors, including ecological interactions, microhabitat diversity [93,94,95,96], predation [88,89], and food availability [87,97]. Therefore, the physiographic features of each area play a central role in shaping the spatial organization of decapod communities in the Ribeira de Iguape River. Species-specific associations with distinct vegetative microhabitats, such as riparian grasses and submerged macrophytes, directly influence community structure. These findings emphasize the ecological importance of habitat complexity in determining species distributions in transitional freshwater environments.

## 5. Conclusions

This study revealed that, despite historical and ongoing human pressures on the Ribeira de Iguape River [13,40,42,43], the region still supports a diverse decapod community composed of freshwater, estuarine, and marine species. The area closest to the river mouth showed the highest species richness and diversity, likely due to its transitional environment and moderate salinity fluctuations. Spatial and seasonal patterns indicated that community structure results from the interaction of abiotic conditions and habitat-related factors. The presence of the non-native *Macrobrachium rosenbergii* suggests an early stage of establishment and reinforces the importance of continued monitoring to assess potential ecological impacts.

Overall, these results reaffirm the ecological importance of freshwater–estuarine transitions and provide a foundation for conservation planning and biodiversity management in the region. The presence of native decapods listed in the Livro Vermelho dos Crustáceos do Brasil further increases the conservation value of this ecosystem and supports the need for long-term monitoring in transitional aquatic environments.

## Figures and Tables

**Table 1:** Overall annual averages and standard deviations of environmental variables by area and season, recorded from February 2014 to January 2015 in the Ribeira de Iguape River, Iguape (SP). (D.O. = dissolved oxygen; Transp. = transparency). Differences were assessed using a generalized linear model (GLM). For variables without a significant area–season interaction, identical lowercase letters in the same column indicate no significant differences. * For salinity, a significant interaction was detected, with Area 3 in spring differing from all other combinations.

	Temperature (°C)	D.O. (mg L^−1^)	Conductivity (µS cm^−1^)	Salinity (ppt) *	pH	Transp. (m)
Area 1	23.7 ± 3.0 a	6.0 ± 1.1 a	84.8 ± 19.7 b	0.04 ± 0.01	7.0 ± 0.4 a	0.5 ± 0.2 a
Area 2	23.5 ± 3.0 a	6.0 ± 1.1 a	91.3 ± 25.0 ab	0.09 ± 0.17	7.0 ± 0.3 a	0.5 ± 0.2 a
Area 3	23.5 ± 2.9 a	5.5 ± 0.9 b	109.6 ± 43.1 a	0.50 ± 1.55	7.0 ± 0.3 a	0.5 ± 0.4 a
Summer	26.5 ± 0.8 a	4.5 ± 0.4 c	80.1 ± 13.7 b	0.04 ± 0.01	6.8 ± 0.3 a	0.3 ± 0.03 b
Autumn	22.2 ± 0.8 b	6.1 ± 0.3 b	109.9 ± 44.5 a	0.05 ± 0.02	6.9 ± 0.1 ab	0.7 ± 0.1 a
Winter	20.3 ± 0.9 c	6.9 ± 0.7 a	80.5 ± 13.1 b	0.04 ± 0.01	7.1 ± 0.2 b	0.6 ± 0.1 a
Spring	25.1 ± 2.8 a	5.8 ± 0.8 b	106.4 ± 32.2 a	0.71 ± 1.78	7.4 ± 1.0 b	0.5 ± 0.1 a

**Table 2:** Species composition, total abundance, size range (minimum and maximum), and total number of individuals by capture method for decapod species (Caridea and Brachyura) recorded in the Ribeira de Iguape River, Iguape (SP), from February 2014 to January 2015. N = total number of individuals. IUCN status: LC = Least Concern; DD = Data Deficient according to Livro Vermelho dos Crustáceos do Brasil [Red List of Brazilian Crustaceans] [58].

Infra-Order	Family	Species	IUCN Status	N	Size (mm)			Collection Methods		
					Min	Max	Avarage ± sd	Box	Minow Trap	Sieve
Caridea	Atyidae	*Potimirim* spp.	LC	20,314	1.5	6.0	3.6 ± 0.9	0	0	20,314
	Palaemonidae	*Macrobrachium acanthurus*	DD	17,332	2.5	35.5	13.9 ± 5.7	3549	330	13,453
		*Macrobrachium carcinus*	DD	54	20.4	73.8	48.7 ± 13.1	0	54	0
		*Macrobrachium olfersii*	LC	1415	1.9	26.0	9.3 ± 4.2	294	82	1039
		*Macrobrachium rosenbergii*	Non- native	2	23.4	27.6	25.3 ± 1.9	0	2	0
		*Palaemon* *pandaliformis*	LC	2647	2.5	8.1	5 ± 0.9	2	0	2645
Brachyura	Sesarmidae	*Armases rubripes*	LC	988	2.2	22.2	13.7 ± 3.0	16	1	971
	Portunidae	*Callinectes bocourti*	LC	43	42.9	110.6	85.8 ± 12.7	1	42	0
		*Callinectes sapidus*	DD	86	15.9	118.0	78.1 ± 23.3	2	78	6
	Ocypodidae	*Minuca mordax*	LC	1		16.0		2	13	0
	Panopeidae	*Panopeus rugosus*	LC	15	25.9	59.8	46.9 ± 9.4	0	0	1

**Table 3:** Composition and abundance of decapod species by sampling area and season from February 2014 to January 2015 in the Ribeira de Iguape River, Iguape (SP).

Species	Areas			Seasons			
	1	2	3	Summer	Autumm	Winter	Spring
*Potimirim* spp.	6687	10,104	3523	2841	2511	6326	8636
*M. acanthurus*	5502	3430	8400	2227	3085	5559	6461
*M. carcinus*	25	27	2	18	19	10	7
*M. olfersii*	452	357	606	304	284	515	312
*M. rosenbergii*	0	1	1	1	0	0	1
*P. pandaliformis*	395	456	1796	302	504	1060	781
*A. rubripes*	104	274	610	291	227	216	254
*C. bocourti*	0	6	37	2	23	2	16
*C. sapidus*	16	26	44	3	36	14	33
*M. mordax*	0	0	1	1	0	0	0
*P. rugosus*	1	2	12	0	4	5	6
Total	13,182	14,683	15,032	5990	6693	13,707	16,507

**Table 4:** Results of multiple linear regressions between environmental variables and abundance for each decapod species. All significant associations observed were positive. (D.O.—dissolved oxygen.).

Species	Predictor Variable	β Coefficient	Standard Error	Adjusted r^2^	F-Value	*p*-Value
*Potimirim* spp.	D.O.	228.22	86.62	0.16	6.94	0.01
*M. acanthurus*	pH	662.41	215.11	0.21	9.48	0.004
*M. olfersii*	Salinity	17.79	7.31	0.14	5.92	0.02
*P. pandaliformis*	Salinity	52.54	20.52	0.15	6.55	0.02
*A. rubripes*	Salinity	17.32	6.04	0.19	8.23	0.017

## Data Availability

The original contributions presented in this study are included in the article. Further inquiries can be directed to the corresponding author.
